# Modernization, collectivism, and gender equality predict love experiences in 45 countries

**DOI:** 10.1038/s41598-022-26663-4

**Published:** 2023-01-14

**Authors:** Piotr Sorokowski, Marta Kowal, Robert J. Sternberg, Toivo Aavik, Grace Akello, Mohammad Madallh Alhabahba, Charlotte Alm, Naumana Amjad, Afifa Anjum, Kelly Asao, Chiemezie S. Atama, Derya Atamtürk Duyar, Richard Ayebare, Daniel Conroy-Beam, Mons Bendixen, Aicha Bensafia, Boris Bizumic, Mahmoud Boussena, David M. Buss, Marina Butovskaya, Seda Can, Antonin Carrier, Hakan Cetinkaya, Ilona Croy, Rosa María Cueto, Marcin Czub, Daria Dronova, Seda Dural, Izzet Duyar, Berna Ertugrul, Agustín Espinosa, Ignacio Estevan, Carla Sofia Esteves, Tomasz Frackowiak, Jorge Contreras Garduño, Karina Ugalde González, Farida Guemaz, Mária Halamová, Iskra Herak, Marina Horvat, Ivana Hromatko, Chin-Ming Hui, Jas Laile Jaafar, Feng Jiang, Konstantinos Kafetsios, Tina Kavčič, Leif Edward Ottesen Kennair, Nicolas Kervyn, Truong Thi Khanh Ha, Imran Ahmed Khilji, Nils C. Köbis, Aleksandra Kostic, Hoang Moc Lan, András Láng, Georgina R. Lennard, Ernesto León, Torun Lindholm, Trinh Thi Linh, Giulia Lopez, Nguyen Van Luot, Alvaro Mailhos, Zoi Manesi, Rocio Martinez, Sarah L. McKerchar, Norbert Meskó, Marija Pejičić, Girishwar Misra, Conal Monaghan, Emanuel C. Mora, Alba Moya-Garófano, Bojan Musil, Jean Carlos Natividade, George Nizharadze, Elisabeth Oberzaucher, Anna Oleszkiewicz, Mohd Sofian Omar-Fauzee, Ike E. Onyishi, Baris Özener, Ariela Francesca Pagani, Vilmante Pakalniskiene, Miriam Parise, Farid Pazhoohi, Annette Pisanski, Katarzyna Pisanski, Edna Ponciano, Camelia Popa, Pavol Prokop, Muhammad Rizwan, Mario Sainz, Svjetlana Salkičević, Ruta Sargautyte, Ivan Sarmány-Schuller, Susanne Schmehl, Anam Shahid, Shivantika Sharad, Razi Sultan Siddiqui, Franco Simonetti, Meri Tadinac, Christin-Melanie Vauclair, Luis Diego Vega, Kathryn V. Walter, Dwi Ajeng Widarini, Gyesook Yoo, Marta Zaťková, Maja Zupančič, Agnieszka Sorokowska

**Affiliations:** 1 University of Wrocław, Wrocław, Poland; 2 Cornell University, Ithaca, NY USA; 3 University of Tartu, Tartu, Estonia; 4 Gulu University, Gulu, Uganda; 5 Middle East University, Amman, Jordan; 6 Stockholm University, Stockholm, Sweden; 7 NUR International University, Lahore, Pakistan; 8 University of the Punjab, Lahore, Pakistan; 9 Westminster College, Salt Lake City, USA; 10 University of Nigeria, Nsukka, Nigeria; 11 Istanbul University, Istanbul, Turkey; 12 THETA Uganda, Kampala, Uganda; 13 University of California, Santa Barbara, USA; 14 Norwegian University of Science and Technology (NTNU), Trondheim, Norway; 15 University of Algiers 2, Algiers, Algeria; 16 Australian National University AU, Canberra, Australia; 17 University of Sétif2, Setif, Algeria; 18 University of Texas at Austin, Austin, USA; 19 Institute of Ethnology and Anthropology, Moscow, Russia; 20 Izmir University of Economics, Izmir, Turkey; 21 Université Catholique de Louvain, Louvain-La-Neuve, Belgium; 22 Yaşar University, Izmir, Turkey; 23 TU Dresden, Izmir, Germany; 24 Pontificia Universidad Católica del Perú, Lima, Perú; 25 Universidad de La República, Montevideo, Uruguay; 26 Católica Lisbon School of Business and Economics, Universidade Católica Portuguesa, Lisbona, Portugal; 27 Unidad Morelia UNAM, Morelia, Mexico; 28 Universidad Latina de Costa Rica, Costa Rica, Costa Rica; 29 Constantine the Philosopher University in Nitra, Nitra, Slovakia; 30 University of Maribor, Maribor, Slovenia; 31 University of Zagreb, Zagreb, Croatia; 32 Chinese University of Hong Kong, Hong Kong, China; 33 University of Malaya, Lumpur, Malaysia; 34 University of Greenwich, London, UK; 35 Aristotle University of Thessaloniki, Thessaloniki, Greece; 36 Palacky University in Olomouc, Olomouc, Czech Republic; 37 University of Ljubljana, Ljubljana, Slovenia; 38 University of Social Sciences and Humanities (VNU-Hanoi), Hanoi, Vietnam; 39 Islamabad Model College for Boys, Islamabad, Pakistan; 40 University of Amsterdam, Amsterdam, The Netherlands; 41 University of Niš, Niš, Serbia; 42 University of Pécs, Pécs, Hungary; 43 University of Urbino Carlo Bo, Urbino, Italy; 44 Vrije Universiteit Amsterdam, Amsterdam, The Netherlands; 45 University of Granada, Grenada, Spain; 46 University of Delhi, Delhi, India; 47 University of Havana, Havana, Cuba; 48 Pontifical Catholic University of Rio de Janeiro, Rio de Janeiro, Brazil; 49 Free University of Tbilisi, Tbilisi, Georgia; 50 University of Vienna, Vienna, Austria; 51 Universiti Utara Malaysia, Sintok, Malaysia; 52 Università Cattolica del Sacro Cuore, Milan, Italy; 53 Vilnius University, Vilnius, Lithuania; 54 University of British Columbia, Vancouver, Canada; 55 University of the State of Rio de Janeiro, Coimba, Brazil; 56 Romanian Academy - Institute of Philosophy and Psychology “C. Rădulescu-Motru”, Bucharest, Romania; 57 Comenius University, Bratislava, Slovakia; 58 University of Haripur, Haripur, Pakistan; 59 Pontificia Universidad Católica de Chile, Santiago, Chile; 60 Institute of Experimental Psychology SAS, Bratislava, Slovakia; 61 DHA Suffa University, Karachi, Pakistan; 62 ISCTE – Instituto Universitário de Lisboa, Lisboa, Portugal; 63 Universitas Prof. Dr Moestopo (Beragama), Jakarta, Indonesia; 64 Kyung Hee University, Kyung Hee, South Korea

**Keywords:** Psychology, Cultural evolution, Anthropology

## Abstract

Recent cross-cultural and neuro-hormonal investigations have suggested that love is a near universal phenomenon that has a biological background. Therefore, the remaining important question is not whether love exists worldwide but which cultural, social, or environmental factors influence experiences and expressions of love. In the present study, we explored whether countries’ modernization indexes are related to love experiences measured by three subscales (passion, intimacy, commitment) of the Triangular Love Scale. Analyzing data from 9474 individuals from 45 countries, we tested for relationships with country-level predictors, namely, modernization proxies (i.e., Human Development Index, World Modernization Index, Gender Inequality Index), collectivism, and average annual temperatures. We found that mean levels of love (especially intimacy) were higher in countries with higher modernization proxies, collectivism, and average annual temperatures. In conclusion, our results grant some support to the hypothesis that modernization processes might influence love experiences.

## Introduction

Many studies have attempted to describe the phenomenon of love. However, only a limited number of scholars have explored love feelings and experiences from a cross-cultural perspective (for notable exceptions, see, e.g.,^1–4^). Even fewer scholars have focused on the observed differences in love levels across cultures (see, e.g.,^5–7^). Yet, such studies provided firm evidence that love varies across cultures^8,9^. Thus, in the present study, we aimed to investigate which cultural and environmental factors might be most pertinent to love experiences.

One of such factors may be the country’s level of modernization^10,11^. Modernization has many meanings, but in the present paper, we define it as a permanent process carried out through reform, education, and innovation, which today means a transition to an industrial and urbanized society^12,13^. This hypothesis has been supported by theories and observations of classical humanists^14,15^ and a few empirical studies. For instance, Belsky et al.^16^ surmised that when children are exposed to harsh physical environments and economic hardships (as in cultures with lower modernization indexes), they tend to exhibit lower levels of romantic love in adulthood. Conversely, when children are provided with sufficient health care, education, and resources (as in cultures with higher modernization indexes), they may experience more intense love and be more emotionally engaged with their partners^17,18^. Thus, it is possible that growing importance of romantic love in adulthood stems from changes in parental emotional investments and better living conditions. Baumard et al.^11^ provided some evidence for such claims. Based on the refined literary analysis of almost 4000 years, Baumard et al. showed that incidences of love increased throughout history with economic development.

Another potential sociocultural factor that might influence love experiences is a classical construct in psychology, namely, individualism-collectivism. From a psychological perspective, collectivism is a value characterized by an emphasis on cohesiveness and prioritization of the group over the self^19,20^. Some studies suggested that level of collectivism influences mate choice and acceptance of arranged marriages^21^, as well as understanding and endorsing the concept of romantic love in romantic relationships^5^. In more collectivistic countries (such as India^22^), love before marriage can be considered a “disruptive element” motivated by selfish interest, which undermines loyalty to family. On the contrary, love is regarded as a basis for marriage among more individualistic Americans^7,23^. Thus, the level of cultural individualism might relate to love patterns in the given society.

Gender equality is the third country-level aspect that is vastly hypothesized to differentiate love experiences across cultures. De Munck and Korotayev^24^ analyzed Rosenblatt’s^25^ data, which consisted of 75 societies, and found that societies in which premarital sex and/or adultery are permitted for both men and women rate romantic love as a more important prerequisite of marriage than do societies in which either one is prohibited. Thus, when women are treated more equally, it might entail their higher agency in choosing with whom they would like to get married (most likely, with someone they love). Furthermore, based on the archival descriptions of traditional societies, the same authors^26^ showed that various factors, possibly related to relationships’ intimacy (e.g., spending leisure time together), significantly predict female status in society. Both analyses are intriguing, but they face similar shortcomings. Authors utilized archival data, which might not reliably represent explicit love levels in analyzed societies. Hence, testing the above hypotheses in contemporary societies that differ on the gender equality continuum could shed more light on the role of gender equality in the love landscape.

To test predictions about cultural differences in love experiences, we conducted a large-scale study of romantic relationships in 45 countries and territories. We tested if country-level modernization indexes, including the Human Development Index (HDI), World Modernization Index (WMI), Gender Inequality Index (GII), and level of collectivism, are related to levels of love across different countries. Many well-known theories of love in the social sciences highlight that love consists of passionate (intense and arousing) and companionate (tender and affective) elements. Such a distinction can also be found in Sternberg’s Triangular Theory of Love^27^.

Sternberg has stated that love consists of intimacy, passion, and commitment. We decided to follow Sternberg’s theory because at least two of its components (i.e., intimacy and passion) perfectly align with our aims. The first component–intimacy—refers to closeness, connectedness, communication, and caring. The second component–passion—pertains to romance, excitement, and physical arousal. Furthermore, as previous studies have shown that environmental variation in temperature induces greater social proximity^28^, influences preferred interpersonal distance^29^, interpersonal touch in close relationships ^30^, and affects emotional expressiveness^31^, we decided to control for each country’s average annual temperature. Because relationship length can affect the intensity of the love components^3^, and the average lengths of relationships varied across the countries we surveyed, we controlled for it in the analyses. We also controlled for sex, as men and women tend to experience love differently^32,33^.

## Results

**Figure 1:**
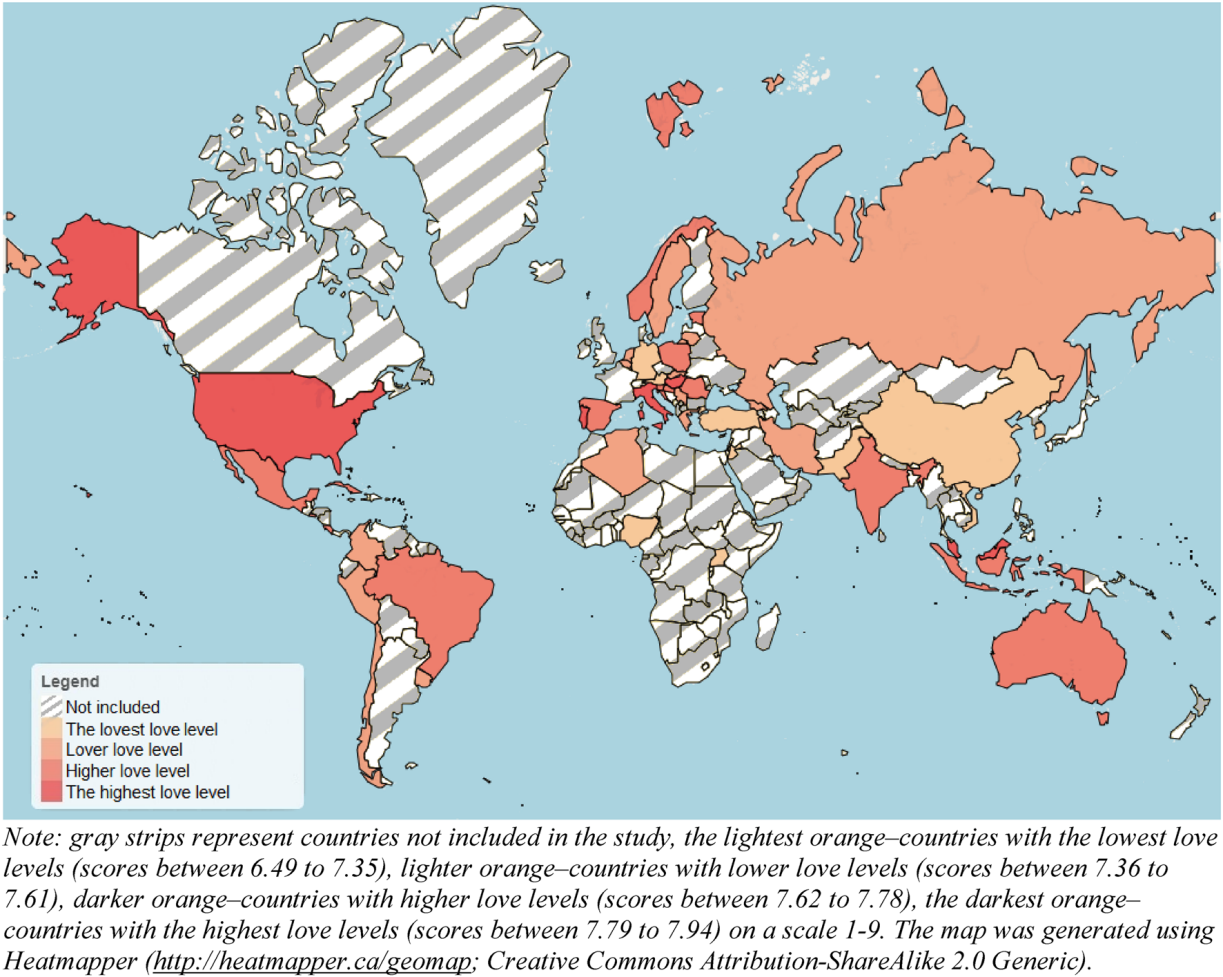
Levels of love (comprised of 45 items from the Triangular Love Scale) across the countries.

Figure 1 presents levels of composite love scores (mean love comprised of 45 items) across countries. The analyses of skewness and kurtosis of the love scales did not provide evidence for the violation of the normality assumptions for large samples. Correlations between variables of interest are presented in Table S1 in the Supplementary Material (SM). Next, we proceeded with multilevel models. Based on the high multicollinearity (VIFs > 5) when computing models with subscales of love as outcome variables and modernization indexes (WMI, GII, and HDI) as predictor variables (raw correlations between these variables ranged from *r* = 0.86 to *r* = 0.93, suggesting that, despite different names, they all might fall under the same umbrella of modernization), we decided to run three separate models for each of the love components. In each of these models, we entered either WMI, GII, or HDI and the remaining variables of interest (country-level collectivism, annual average temperatures, and participants’ sex and relationship length) as predictor variables. The outcome variables were the composite TLS-45 score (a mean of 45 items) and a composite score (a mean of 15 corresponding items) of each of the love subscales (i.e., intimacy, passion, and commitment). Here, we present the results of the models that explained the most variance (see Table S2 in the Supplementary Material for a comparison of explained variance), that is, models with HDI (see Table 1).

**Table 1:** Results of the multilevel linear models regressing love components (i.e., STLS-45, intimacy, passion, and commitment) on countries’ levels of Human Development Index (HDI), Collectivism level, annual average temperature, and participants’ sex and length of relationship (in months), with participants nested within countries.

	STLS-45				Intimacy				Passion				Commitment			
	*β*	*SE*	*95% CI*	*p*	*β*	*SE*	*95% CI*	*p*	*β*	*SE*	*95% CI*	*p*	*β*	*SE*	*95% CI*	*p*
**Fixed effects**																
HDI^a^	0.233	0.043	[0.043, 0.148]	< 0.001***	0.312	0.046	[0.046, 0.221]	< 0.001***	0.143	0.049	[0.049, 0.047]	0.004**	0.215	0.039	[0.039, 0.139]	< 0.001***
Collectivism^b^	0.074	0.033	[0.033, 0.010]	0.023*	0.094	0.035	[0.035, 0.026]	0.007**	0.054	0.037	[0.037, − 0.018]	0.145	0.059	0.029	[0.029, 0.001]	0.045*
Temperature^c^	0.099	0.035	[0.035, 0.031]	0.004**	0.084	0.037	[0.037, 0.012]	0.023*	0.118	0.039	[0.039, 0.041]	0.003**	0.077	0.031	[0.031, 0.016]	0.014*
Sex^d^	− 0.016	0.011	[0.011, − 0.038]	0.152	0.027	0.011	[0.011, 0.006]	0.012*	− 0.042	0.011	[0.011, − 0.064]	< 0.001***	− 0.022	0.011	[0.011, − 0.043]	0.055
Relationship length^e^	− 0.01	0.011	[0.011, − 0.032]	0.363	− 0.025	0.011	[0.011, − 0.046]	0.021*	− 0.076	0.011	[0.011, − 0.098]	< 0.001***	0.081	0.011	[0.011, 0.059]	< 0.001***
**Random effects**																
Variance	0.030				0.035				0.042				0.023			
Variance *SD*	0.174				0.188				0.205				0.150			
ICC	0.035				0.043				0.045				0.026			
Pseudo *r*^2^	0.032				0.065				0.021				0.035			
df_residuals_	7532				7532				7524				7459			
Deviance	20,192				19,720.7				20,612.6				20,081.5			

*p < 0.05, **p < 0.01, ***p < 0.001. ^a^Human Development Index, ^b^Collectivism-in-group favoritism, ^c^Temperature–Average annual temperatures, ^d^Participants’ sex with men as a reference group, ^e^Participants’ relationship length in months.

The results showed that HDI and country-level average annual temperatures were positively related to the STLS-45, intimacy, passion, and commitment, while country-level collectivism was positively related to the STLS-45, intimacy, and commitment. That would mean that inhabitants of more modernized countries with higher average annual temperatures would, on average, experience higher levels of all love components. Furthermore, more intimacy and commitment would be experienced by those from more collectivistic countries. We also found evidence that, controlling for other factors in the model, women had a higher mean level of intimacy but a lower mean level of passion than men. Furthermore, the longer the relationship, the lower the mean level of experienced intimacy and passion, but the higher the mean level of commitment.

A similar pattern of results was yielded in the case of the two other proxies of countries’ modernization levels. World Modernization Index was positively and Gender Inequality negatively related to the STLS-45 (*β* = 0.181, *p* < 0.001, pseudo *r*^2^ = 0.018*, β* = -0.138, *p* < 0.001, pseudo *r*^2^ = 0.011*,* respectively), intimacy (*β* = 0.264, *p* < 0.001, pseudo *r*^2^ = 0.046, *β* = -0.178, *p* = 0.002, pseudo *r*^2^ = 0.024, respectively), and commitment (*β* = 0.169, *p* = 0.007, pseudo *r*^2^ = 0.022, *β* = -0.142, *p* < 0.002, pseudo *r*^2^ = 0.019, respectively), see Tables S3–S4 in the SM for detailed results. We have also tested the above models with participants’ age as a control variable. However, because participants’ age and relationship length were highly correlated (*r* = 0.83), we did not introduce age simultaneously but rather interchangeably with relationship length. The patterns of results between love components and cultural and environmental variables remained the same, except for country’s collectivism level, which ceased to be significantly related to intimacy (see Tables S5–S7 in the Supplementary Material).

As we observed stronger effects for intimacy than passion, in an explorative vein, we also tested for models with passionate love (i.e., passion to intimacy ratio) as an outcome variable. We found that the amount of passion to intimacy ratio was lower in countries with higher modernization indexes (see Tables S3, S4 and S8 in the SM for details).

**Figure 2:**
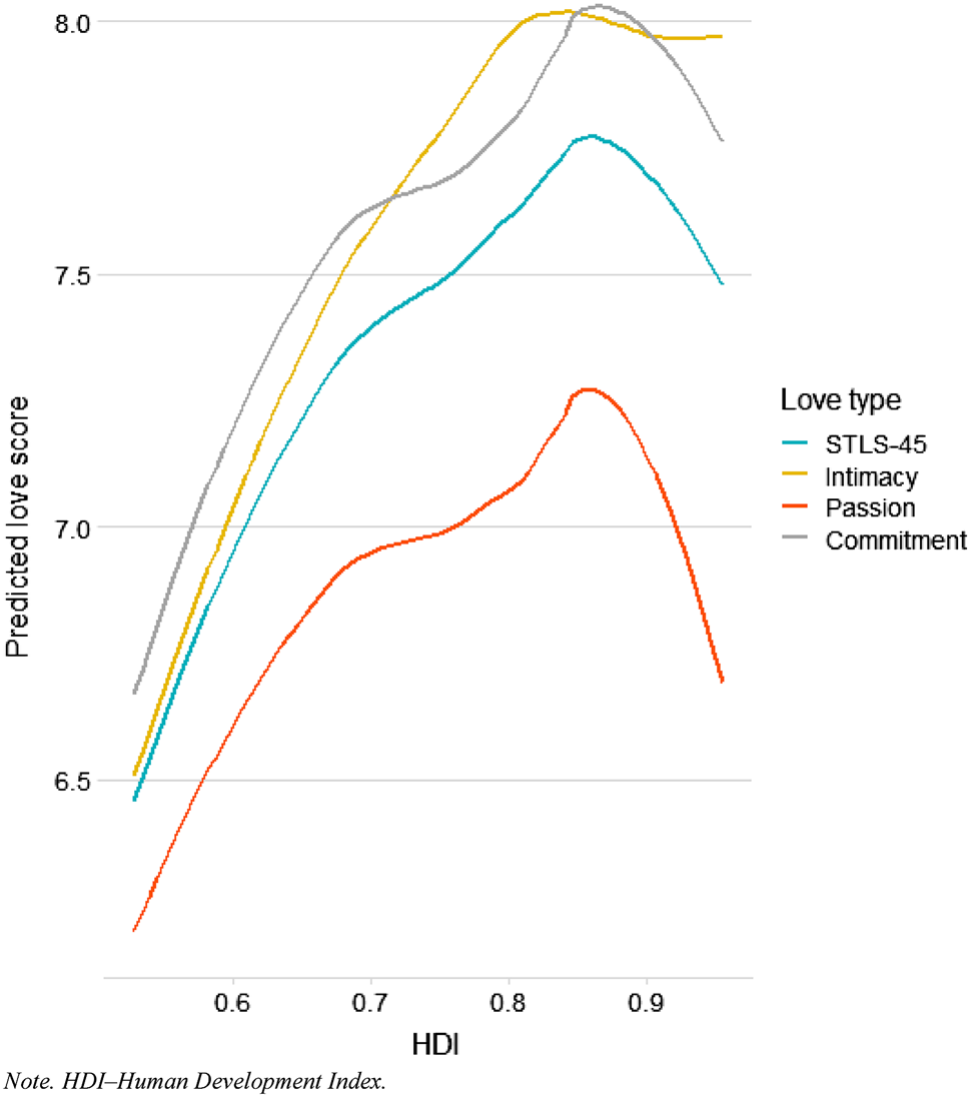
A graphical representation of the non-linear relationship between predicted love scores and Human Development Index (controlling for country’s collectivism, average annual temperature, and participants’ sex and relationship length).

In the last step, we tested for non-linear relationships between the outcome and predictor variables. As became evident from the scatterplots (see Fig. 2 and Figs. S1–S4 in the SM), after a certain threshold of modernization (e.g., ~ 0.85 in the case of HDI), mean levels of STLS-45, passion, and commitment tended to decrease. These conclusions were further confirmed by the results of the multilevel models, which included the squared term of modernization indexes (see Tables S9–S11 in the SM for detailed results).

## Discussion

Many descriptive works show how love experiences may change with various levels of modernization^34,35^. Other study supported such claims based on the analysis of incidences of love in narrative fiction throughout centuries^11^. However, based on quantitative, cross-cultural data, our study is the first to provide evidence on how love experiences vary concerning different levels of human development and modernization indexes. We observed that, in general, participants from countries with higher (compared with countries with lower) levels of HDI, World Modernization Index, and gender equality experienced more love with their partners, controlling for participants’ sex, relationship length, countries’ average annual temperatures, and collectivism level. However, after reaching a certain, relatively high threshold of modernization (e.g., in the case of HDI—0.85), mean love levels tend to drop. Overly simplifying, we can conclude that more modernized countries have a higher level of all love subscales (though this effect is more pronounced for intimacy than passion), but the highest levels of modernization do not promote intense love experiences.

Furthermore, the results provided tentative evidence that higher mean levels of intimacy and commitment are positively related to countries’ level of collectivism. It is especially interesting, considering that previous studies highlighted the importance of romantic love in relationships established in more individualistic cultures^7,23,26^ as opposed to more collectivistic cultures, in which, historically, arranged rather than love marriages have been more prevalent^36,37^. On the other hand, collectivistic values promote a more relational view of romantic relationships^38^. Thus, individuals from more collectivistic countries might be more altruistic towards their partners^5,39^, which could naturally lead to more intimate and stronger bonds between the lovers^40^. However, the observed relationships ceased to be significant when controlling for participants’ age. Also, we did not observe any links between passion level and country’s collectivism index. Considering the most recent cultural changes in collectivistic values in various countries^41^, future studies could investigate whether individual levels of collectivistic beliefs might be more related to experiences love than country-levels of collectivism.

Relatively modest relationships between modernization indexes and passion suggest that passion is rather stable across different modernization levels, and that what carries the relationship between the passionate love (i.e., passion to intimacy ratio) and modernization indexes is higher intimacy in countries with higher modernization indexes. A growing body of research provides evidence for biological antecedents of passion and its role in reproduction (see, e.g.,^42–44^), and thus, the stability of passionate experiences across various countries seems unsurprising. Furthermore, in line with previous works^3,44,45^, we observed lower levels of passion and intimacy, and higher levels of commitment among participants with longer relationship duration.

However, questions regarding the mechanisms behind the observed patterns of changes in intimacy/commitment are more challenging to answer. The simplest explanation might be that people from countries with higher modernization indexes tend to emphasize the friendship aspect of relations with their partners^46^. Indeed, some studies provided evidence that individuals from countries with higher modernization indexes expect love to be based on mutual attraction and emotional closeness^31,47^. Apart from the environmental and economic factors already tackled in the introduction (i.e., the growing importance of romantic love in adulthood possibly resulting from changes in parental emotional investment and better living conditions^11,16,48,49^), we can also hypothesize other possible explanations.

For instance, cultural changes stem from processes of democratization, emancipation of love^34,50,51^, gender shifts, and increasing gender equality^52,53^. Because love becomes increasingly dependent on the capitalist market, such processes may also promote specific love patterns (that is, more intimate love but not that much of sexual love^47,54^). We might also consider social changes in terms of cultural perception of reproduction or, in general, postponed reproduction in countries with higher modernization indexes^55,56^. Several of these factors may be responsible for the observed increasing role of intimacy in societies with higher modernization indexes. Future research should focus on disentangling modernization components, which would shed more light on which specific factors drive the observed patterns.

Furthermore, we observed a distinctive drop in the mean levels of love among participants from countries that reached a relatively high level of modernization (e.g., in the case of HDI, the threshold was 0.85). This suggests that, although country’s economic development generally promotes more intense love experiences, reaching a certain developmental point might reverse these beneficial love effects. Such hypotheses have been indirectly laid by ethologists studying animal behaviors^57,58^. For instance, in a classical study, Calhoun^57^ observed that mice thrived when granted unlimited access to all necessary resources. However, mice started to lose interest in mating and reproduction when the situation was too good for too long. We can only speculate to which extent such an animal model might apply to humans.

Interestingly, research on the role of temperature in social interactions evokes heated discussions. We found some evidence that a country’s average temperature is positively related to love experiences. When controlling for other factors, we found that participants from countries with higher annual temperatures reported higher levels of love (though this effect was the strongest for passion). However, raw correlations showed the opposite patterns, meaning that participants from countries with higher temperatures experienced lower intimacy and commitment levels. As results of previous studies also yielded contradictory conclusions^28,29^, future investigations might attempt to deepen our understanding of the role of climate and temperature on humans’ feelings and behaviors.

**Figure 3:**
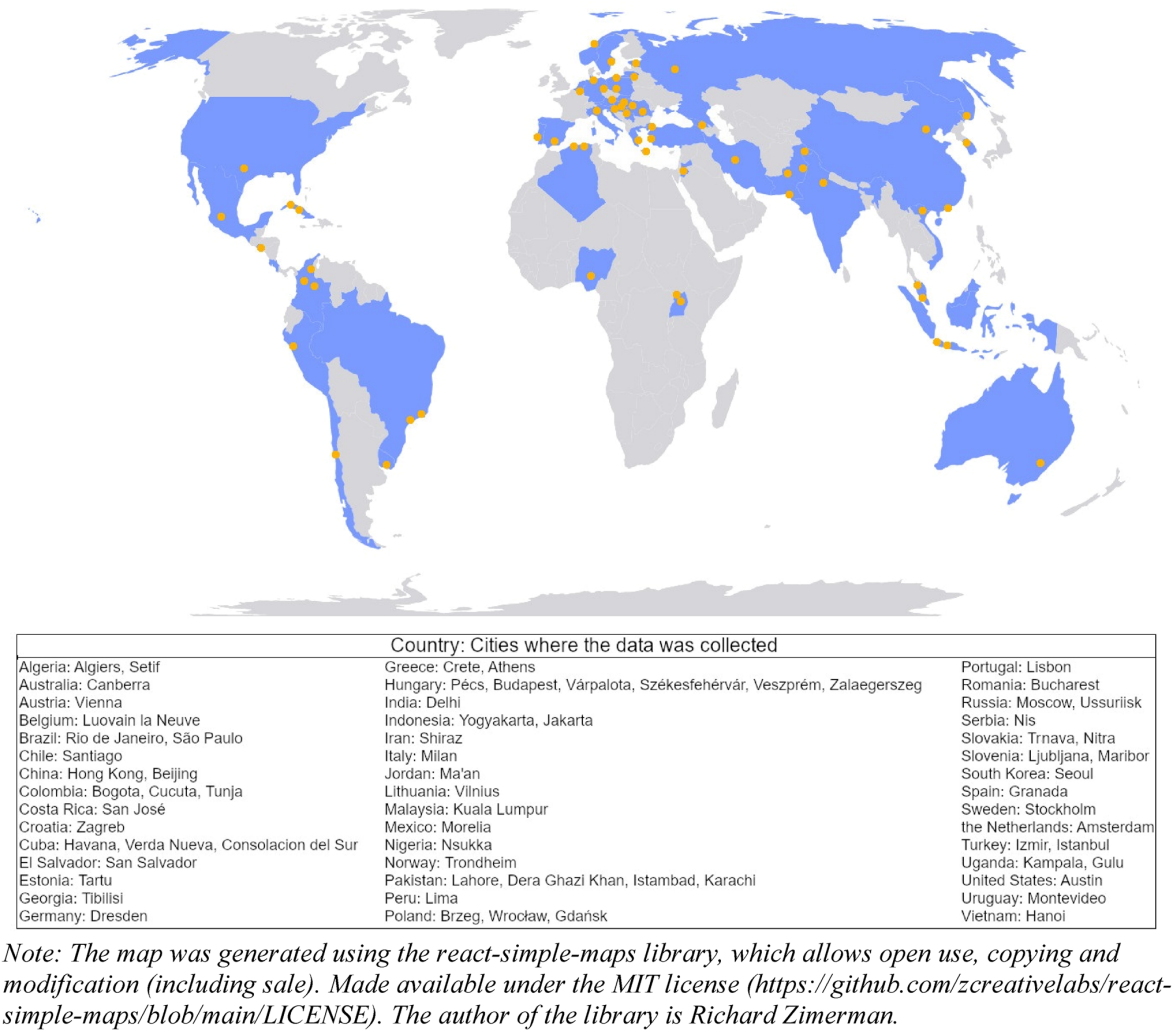
Locations of data collection. Countries (in blue) with corresponding study sites (cities in orange).

Although the current study sheds new light on the cultural evolution of love, it is not free of limitations. First, despite recruiting a relatively large number of participants from various cultures, one needs to bear in mind that the studied sample was not representative of any of the 45 countries. Moreover, our participants were relatively well-educated and from urban areas (see Fig. 3), which makes them even less representative of less modernized countries. Second, although we used one of the most famous love scales, the Triangular Love Scale^27^, the scale has been criticized for high correlations between love components^59,60^. Furthermore, the TLS might not reliably distinguish participants with high levels of love^61^. As love measures are not perfectly correlated (their correlations tend to vary from 0.00 to even 0.83, see^62,63^), it would be interesting to test the present results' robustness using different love measures. Third, we have focused on cultural and environmental variables at the country-level. Future studies could investigate whether individual-level factors identified in the present study contribute to love experiences in a similar vein. There is some evidence that, for instance, psychological collectivism might impact love patterns differently^64^.

In conclusion, our study—one of the largest studies on cross-cultural differences in love experiences to date—provided evidence that, at least at the beginning of the twenty-first century, love is a near universal human experience. The results of the present investigation offer valuable insight into cultural and environmental factors related to countries’ variability of love experiences. Although our research is correlational and no causal conclusions can be made, one may hypothesize that cultural changes in the level of a country’s modernization index may affect patterns of love (i.e., may increase experiences of intimacy and commitment). More studies conducted in countries with lower levels of modernization using a longitudinal design might address this hypothesis.

Our study showed that love experiences differ across cultures. The results corroborate previous research findings on similarities and differences in how people chose their love partners^65^ and how their choices affect their relationship satisfaction^66,67^. However, as a concluding remark, we would like to highlight that we believe there is no better or worse way to experience love. On the contrary, understanding different love patterns may be crucial in studying the vast phenomenon of love. Exploring how love differs across cultures may result in identifying the love hardships of couples from different cultural backgrounds, which may, inter alia, promote developing more accurate and effective strategies in couple counseling.

## Methods

### Ethical statement

All participants gave written informed consent to participate. The study protocol was approved by the Institutional Ethics Committee at the Institute of Psychology at the University of Wrocław. Furthermore, all methods were performed in accordance with the guidelines of the Declaration of Helsinki. The other co-authors acquired ethical consent at their institutions when necessary. Russian data were collected in line with the state assignment # 01201730995 of the Institute of Ethnology and Anthropology (MB and DD).

### Participants

Data for the present study were obtained from our published dataset^3^, which reported a large-scale study of sexual and romantic relationships conducted in 45 countries and territories in 2018 (see, e.g.,^3,30,68^).

Only participants 18 years old or above were invited to participate in the study. Approximately half of the sample was recruited from outside of the university community. The original sample comprised 11,422 participants from 45 countries. Herein, we analyze data only from participants who reported being in a relationship (i.e., dating, engaged, or married) and completed all information about their relationship (i.e., type and length). As eight countries had small sample sizes (Colombia *n* = 22, El Salvador *n* = 42, Germany *n* = 57, Greece *n* = 49, Indonesia *n* = 23, Iran *n* = 22, Jordan *n* = 28, Nigeria *n* = 36), and the sex disproportion was substantial (i.e., below 40% of men or women) in 17 countries (Austria, Chile, Colombia, El Salvador, Germany, Greece, Iran, Italy, Nigeria, Peru, Portugal, Slovakia, Spain, Uganda, USA, Uruguay, Vietnam), we recruited additional participants so that at least 70 individuals would represent each country and so that the proportion of each sex would be no more imbalanced than 40% to 60%. New participants (18 years or older, currently in a relationship: dating, engaged, or married) were recruited in two ways: first, by posting the invitation to participate in the study in various groups on social media (*n* = 134) and with the use of an outsourcing company (*n* = 462). We increased the sample size to increase the number of analyzed countries and to ensure that the observed relationships were not spurious due to the impact of variability stemming from the abovementioned reasons. Importantly, increasing the sample size (*n* = 596) did not change any of the main results in our study (see Tables S12–S14 for results of the analyses based on the original dataset). All additional participants were distinguished in the database (which can be found in the Supplementary Material, under the link: https://figshare.com/s/25d3cc3ec48e6b5a6d64). The final sample consisted of 9,474 participants (56% women) from 45 countries (mean age = 30.53, *SD* = 10.95), with average relationship length of 87.46 (*SD* = 104.56) months. Detailed information about the participants and concerning the countries can be found in the SM (Table S15). Figure 3 shows an overview of countries and sites where the data were primarily collected.

### Procedure

The data from the large-scale study were collected simultaneously across all study sites. We exercised great care to ensure similar recruitment methods in all countries. Before the data collection, each collaborating researcher got acquainted with detailed study protocols. In countries where English was not a primary language, collaborating researchers performed a forward-back translation by separate translators when possible^69^. Participants were not compensated for their participation in the study. The study was conducted before the COVID-19 pandemic. After providing informed, written consent to participate in the study, participants were given a set of questionnaires, including the current love scale and several unrelated questionnaires about romantic relationships (see, e.g.,^68,70^). Additional online data were collected in 2021 by the two first authors (via social media and the outsourcing company).

### Variables

In the present study, we used the 45-item version of the Sternberg's Triangular Love Scale (STLS)^27^. It consists of 15 items about intimacy (e.g., *I share deeply personal information about myself with*…), 15 items about passion (e.g., *Just seeing*… *excites me*), and 15 items about commitment (e.g., *I have confidence in the stability of my relationship with…*). Answers range from 1—*Not at all*, to 9—*Extremely*. The scale was highly reliable: Cronbach’s α for the STLS-45 = 0.97, *α* = 0.94 for intimacy, *α* = 0.94 for passion, and *α* = 0.95 for commitment. A detailed description of the equivalence of invariance across countries can be found in Sorokowski et al.^3^.

To test the level of modernization in each country, we used the World Modernization Index (WMI)^71^. This measure was based on World Development Indicators (published by World Bank) and Statistical Yearbook (published by, inter alia, United Nations). The World Modernization Index reflects the composite levels of modernization in the economy, society, knowledge, and environment. The WMI consists of First Modernization, a classical modernization index that typically features industrialization, urbanization, and democratization, and Second Modernization, a new modernization that typically features knowledge, innovation, and transmission^71^. In the present study, we used an integrated modernization index, a combination of these two indexes (i.e., First Modernization and Second Modernization).

As scholars use various proxies to control for the level of modernization across countries^72–75^, we additionally tested our hypothesis using the Human Development Index, obtained from the United Nations Development Programme^76^.

We used Gender Inequality Index (GII), which measures gender inequality in several contexts (e.g., inequalities in reproduction health or force participation and labor market rate of men and women over 15 years). The data on GII was obtained from United Nations Development Programme^76^.

Collectivism (in-group favoritism) levels were received from van de Vliert^77^. This scale highly correlates with the classical Hofstede individualism-collectivism scale but, contrary to the Hofstede scale, contains data on all countries included in the present analyses.

The data on the annual average temperature of each country were obtained from the Tyndall Centre for Climate Change Research^78^.

### Statistical analyses

In the first step, mean levels for the STLS-45 (45 items), intimacy (15 items), passion (15 items), and commitment (15 items) across participants were calculated. In the second step, the normality assumptions of love subscales were investigated, adhering to commonly recommended thresholds for large sample data (i.e., |2| for skewness and |7| for kurtosis^79^).

Pearson correlations were then computed. Next, country-level variables were grand-mean centered and individual-level variables were group-mean centered. Further, multilevel analyses with a maximum likelihood estimator were conducted. Participants were nested within countries to account for the non-independence between the inhabitants of the same geographical territories. In these models, STLS-45, intimacy, passion, and commitment, were introduced as outcome variables and World Modernization Index, Gender Inequality Index, Human Development Index, Collectivism level, and annual average temperature, participants’ sex and length of relationship (in months), as predictor variables. Next, the amount of multicollinearity was investigated using the Variance Inflation Factor (VIF), and models fit with the amount of explained variance. The recommended guidelines were adhered to, that is, VIF > 5 indicating possible issues with collinearity^80,81^. In the final step, visual representations of non-linear relationships between the outcome variables and predictor variables were inspected. All the analyses were performed in R (version 4.2.0).

## Data Availability

All data and the Supplementary Material can be found under the link: https://figshare.com/s/25d3cc3ec48e6b5a6d64.
